# Modeling neurodegeneration in *Caenorhabditis* *elegans*

**DOI:** 10.1242/dmm.046110

**Published:** 2020-10-26

**Authors:** Kim A. Caldwell, Corey W. Willicott, Guy A. Caldwell

**Affiliations:** 1 Department of Biological Sciences, The University of Alabama, Tuscaloosa, AL 35487, USA; 2 Departments of Neurobiology, Neurology, Center for Neurodegeneration and Experimental Therapeutics, and Nathan Shock Center of Excellence in the Basic Biology of Aging, University of Alabama at Birmingham, Birmingham, AL 35294, USA

**Keywords:** Aging, Behavior, Genetics, Modeling, Phenotype, Proteostasis

## Abstract

The global burden of neurodegenerative diseases underscores the urgent need for innovative strategies to define new drug targets and disease-modifying factors. The nematode *Caenorhabditis elegans* has served as the experimental subject for multiple transformative discoveries that have redefined our understanding of biology for ∼60 years. More recently, the considerable attributes of *C. elegans* have been applied to neurodegenerative diseases, including amyotrophic lateral sclerosis, Alzheimer's disease, Parkinson's disease and Huntington's disease. Transgenic nematodes with genes encoding normal and disease variants of proteins at the single- or multi-copy level under neuronal-specific promoters limits expression to select neuronal subtypes. The anatomical transparency of *C. elegans* affords the use of co-expressed fluorescent proteins to follow the progression of neurodegeneration as the animals age. Significantly, a completely defined connectome facilitates detailed understanding of the impact of neurodegeneration on organismal health and offers a unique capacity to accurately link cell death with behavioral dysfunction or phenotypic variation *in vivo*. Moreover, chemical treatments, as well as forward and reverse genetic screening, hasten the identification of modifiers that alter neurodegeneration. When combined, these chemical-genetic analyses establish critical threshold states to enhance or reduce cellular stress for dissecting associated pathways. Furthermore, *C. elegans* can rapidly reveal whether lifespan or healthspan factor into neurodegenerative processes. Here, we outline the methodologies employed to investigate neurodegeneration in *C. elegans* and highlight numerous studies that exemplify its utility as a pre-clinical intermediary to expedite and inform mammalian translational research.

## Abstract (precis)

**Summary:** While unsurpassed as an experimental system for fundamental biology, *Caenorhabditis elegans* remains undervalued for its translational potential. Here, we highlight significant outcomes from, and resources available for, *C. elegans-*based research into neurodegenerative disorders.

## Introduction

Neurodegenerative diseases represent a significant and growing burden globally and the identification of factors that reduce their impact necessitates considerable research effort. Undoubtedly, rodent models have expanded our understanding of neurodegenerative disorders over the past few decades. However, a range of phenotypic variations can manifest in humans; therefore, it seems prudent to use diverse model organisms to hasten research into causes and cures for neurodegenerative diseases. In this At a Glance article, and the accompanying poster, we survey *Caenorhabditis elegans* as a model for neurodegenerative disease research. Many consider this nematode roundworm to be the most completely understood animal on the planet. Therefore, given the sense of urgency to accelerate discovery, the assays, molecular tools and genetic strategies developed through years of collaborative and collective effort in the worm community should be seriously considered within the arsenal for eradicating neurodegenerative diseases from the human population.

**Figure:**
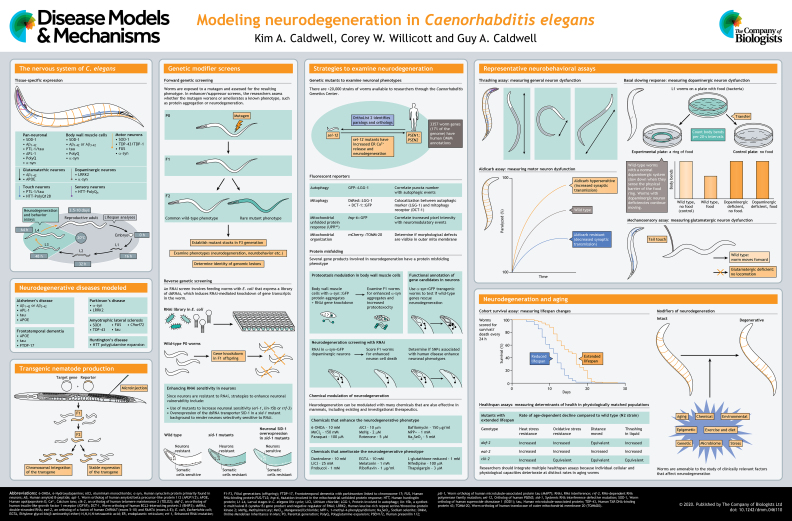

Despite the lack of evolutionary complexity, *C. elegans* shares many conserved molecular pathways and cellular mechanisms with mammals, thus allowing for comparative studies. OrthoList 2 is a searchable compendium of all orthologs and paralogs between *C. elegans* and humans and includes 7943 genes, or ∼41% of the *C. elegans* protein-coding genome (Shaye and Greenwald, 2011; Kim et al., 2018a). Moreover, 3357 worm genes possess human orthologs with Online Mendelian Inheritance in Man (OMIM) annotations; this is 17% of the worm genome (Kim et al., 2018a; http://ortholist.shaye-lab.org/). This conservation provides great potential for modeling human genetic disease. For example, many *C. elegans* neuronal genes are expressed in comparable mammalian cell types (Hobert, 2013).

While mammalian nervous systems consist of billions of neurons, the adult *C. elegans* hermaphrodite has ∼300 neurons throughout its body, thereby reducing the complexity and increasing the accuracy of neuronal analyses (Cook et al., 2019). The major functional components of mammalian synaptic transmission, such as neurotransmitters, receptors, transporters and ion channels, are conserved in *C. elegans*. Based on differences in function, morphology and connectivity, the neurons of this animal have been sorted into separate classes: mechano-, chemo- and thermo-sensory neurons, interneurons, motor neurons and neuromuscular junction components, among others. Importantly, all neuron types can be further subcategorized by neurotransmitter biosynthesis and activity; neuronal signaling molecules include glutamate (Li et al., 1997), GABA (McIntire et al., 1993), acetylcholine (Alfonso et al., 1994), dopamine (Sulston et al., 1975), serotonin and octopamine (Horvitz et al., 1982), as well as neuropeptides (Li and Kim, 2008). Stereotypical behavioral phenotypes are associated with distinct neuronal subclasses (see poster, ‘Representative neurobehavioral assays’). The *C. elegans* neuronal circuitry has also been fully mapped since the 1980s (White et al., 1986), and researchers recently updated the compendium of the neuronal connectomes of both the male and hermaphrodite using state-of-the-art technologies (Cook et al., 2019). Moreover, the constituent gene expression patterns, as discerned by the collective efforts of researchers in the CeNGEN Project, facilitate enhanced understanding of neuronal signaling at a level of detail simply not yet possible in other species (Hammarlund et al., 2018). Furthermore, because *C. elegans* is anatomically transparent, neurons are easily visualized by expressing fluorescent proteins in live animals to study neuronal properties throughout development and over time.

## Genetic models of neurodegeneration

*C. elegans* is informative for basic studies of neuronal function. Perhaps more impressively, it can be effectively harnessed to rapidly and cost effectively identify gene targets and drug candidates that modify neurodegenerative processes. In contrast, rodent neurodegeneration studies are time consuming and economically challenging. Despite the abundance of cellular and animal models to study neurodegenerative disease, we contend that *C. elegans* has been underutilized with respect to its predictive capacity for translational biology. In this At a Glance article, we describe common forms of neurodegeneration modeled in *C. elegans* (see poster, ‘Neurodegenerative diseases modeled’), including Alzheimer's disease (AD), amyotrophic lateral sclerosis (ALS), frontotemporal dementia (FTD), Huntington's disease (HD) and Parkinson's disease (PD). We discuss the genetic and environmental modulators that share common pathways leading to cellular malfunction and eventually neurodegeneration within the context of these *C. elegans* human disease models. There are additional models of these neurodegenerative diseases that are not covered within the article, and we apologize for this omission due to space constraints. Furthermore, we have largely excluded discussions of any models in which neurodegeneration was not an endpoint phenotype evaluated.

### AD

Two defining characteristics of AD are the presence of insoluble amyloid β-peptide (Aβ) plaques and of tau-associated neurofibrillary tangles in the brain. In mammals, Aβ is accumulated into extracellular plaques, followed by endocytic uptake of these neurotoxic oligomers. This process induces tau phosphorylation and aggregation into neurofibrillary tangles. Aβ accumulation is an outcome of sequential enzymatic processing of the human amyloid precursor protein (APP) by membrane-bound secretase enzymes mediated by the subcomponent PS1 and PS2 presenilin proteins. Although the product of the worm *sel-12* gene provided some of the first evidence for presenilins as functional subunits of human γ-secretase (Levitan and Greenwald, 1995), the *C. elegans* genome lacks both a clear β-secretase ortholog and an ortholog of APP that would be processed to yield Aβ peptides. The worm APL-1 protein is an ortholog of the human amyloid beta precursor-like proteins 1 and 2 (APLP1 and APLP2), and has 71% sequence similarity to the intracellular domain of APP, but completely lacks an Aβ domain. Thus, AD modeling in *C. elegans* has primarily focused on the transgenic expression of mature human Aβ and tau (Griffin et al., 2017). Nevertheless, single-copy insertion and expression of human APP results in neurodegeneration and neurobehavioral dysfunction in *C. elegans* (Yi et al., 2017)*.* Likewise, although *apl-1* is essential for viability, overexpression of *apl-1* yields pleiotropic phenotypes that include neuronal deficits (Hornsten et al., 2007; Ewald et al., 2012). Owing to spatial constraints, the AD section of this article will focus on Aβ modeling, while the ALS section will describe tau expression in *C. elegans*. However, we direct the reader to studies in which transgenic nematodes expressing human tau have been generated and include the fine work of several groups with interests in AD modeling (Kraemer et al., 2006; Ayyadevara et al., 2016; Pir et al., 2016; Wang et al., 2018; Fang et al., 2019).

Modeling AD in *C. elegans* has largely been based on the ‘amyloid cascade hypothesis’ (Hardy and Selkoe, 2002). Here, the Aβ_1-42_ peptide is thought to be the most toxic species generated by the cleavage of APP. The original and most commonly used *C. elegans* AD model was a result of pioneering research by Chris Link (1995). In this model, and related variants, the Aβ peptide is tagged with a secretion signaling sequence that is expressed from the *unc-54* promoter in the body wall muscles, where it causes paralysis, thereby allowing for quantitative analysis of modulators. Aβ_1-42_-mediated neurodegeneration has been studied primarily in mechanosensory and in glutamatergic neurons, which are commonly susceptible to hyperexcitability and are clinically associated with the early stages of degeneration in AD (Palop and Mucke, 2009, 2016). Wild-type *C. elegans* have five glutamatergic neurons in their tails. Models of AD that overexpress GFP-fused human Aβ_1-42_ specifically in these neurons under the *eat-4* glutamate transporter promoter demonstrate progressive, age-dependent neurodegeneration (Treusch et al., 2011). Researchers utilized a yeast model expressing Aβ_1-42_ targeted to the secretory pathway to identify genetic modifiers of Aβ toxicity. This genome-wide screen revealed 23 suppressors and 17 enhancers of toxicity, with 12 of these having human homologs. These modifiers were then tested in *C. elegans* to determine their neurotoxicity. Phosphatidylinositol-binding clathrin-assembly protein (*PICALM*) protected against Aβ neurotoxicity, which was further verified in primary rat cortical neurons and corroborated the human genetic data implicating this gene in AD (Treusch et al., 2011). In a separate study, a high-throughput compound screen in yeast and *C. elegans* models of AD identified a class of metal-binding compounds that were neuroprotective through the degradation of Aβ (Matlack et al., 2014). Further investigation showed that these small molecules were related to clioquinol, which was previously found to reduce Aβ toxicity in mouse models (Cherny et al., 2001). Using the same transgenic *C. elegans* Aβ_1-42_ model, Lu et al. (2014) showed that Wnt/β-catenin signaling attenuated glutamatergic neuron loss through regulation of *spr-4*, a homolog of the stress regulatory transcription factor *REST*.

The *ε2* isoform of apolipoprotein E (*APOE*) has been associated with reduced risk for developing AD, whereas the *APOEε4* allele increases risk, with *APOEε3*, the most frequent isoform, being neutral (Spinney, 2014). Our group developed a suite of transgenic *C. elegans* models expressing human *APOE* alleles, with and without Aβ_1-42_ (Griffin et al., 2019). Co-expressing *APOEε2* with Aβ protected glutamatergic neurons from degeneration and restored normal mechanosensory behavior. In contrast, the *APOEε3* worms displayed an intermediate phenotype, and expression of *APOEε4* did not protect from Aβ neurotoxicity. In the absence of Aβ, the *APOE* alleles did not alter glutamatergic neuron function. These allele-specific neuroprotective effects could be modulated in response to endoplasmic reticulum (ER)-associated calcium changes, both pharmacologically and via RNA interference (RNAi) knockdown. Moreover, lifespan was reduced in *C. elegans* expressing Aβ and *APOEε4*; it was rescued by *APOEε2* and *APOEε3* alleles (Griffin et al., 2019). Thus, transgenic *C. elegans* lines recapitulate the established clinical profile of *APOE* polymorphism-associated impact on neurodegeneration, which can be used to discern new mechanistic insights for AD.

### ALS and FTD

ALS is a neurodegenerative disorder that affects the upper and lower motor neurons, causing progressive muscle weakness. More recently, FTD, a dementia syndrome that can present with changes in behavior or language dysfunction, has become recognized in many ALS patients (Caga et al., 2019; Zucchi et al., 2019). Most ALS is sporadic, with only ∼10% of cases displaying a familial inheritance pattern. A causative mutation has been identified in ∼80% of these kindreds; notably, some of these same mutations also lead to sporadic ALS and many are autosomal dominant (Al-Chalabi et al., 2017). *C. elegans* has been used to model the four most common ALS-causing mutations in C9orf72-SMCR8 complex subunit (*C9orf72*), superoxide dismutase (*SOD1*), TAR DNA-binding protein (*TARDBP*) and RNA-binding protein FUS/TLS (*FUS*), as well as a mutation associated with FTD [microtubule-associated protein tau (*MAPT*)].

Mutations in *C9orf72*, where a hexanucleotide repeat (GGGGCC) within the first intron of *C9orf72* undergoes expansion, are responsible for 10-15% of familial ALS cases (van Damme et al., 2017). The mechanism associated with this expansion remains under investigation. One hypothesis is that the translation of these repeats is AUG independent. This example of repeat-associated non-AUG (RAN) translation (Cleary and Ranum, 2017) can result in five separate dipeptide-containing proteins from the GGGGCC repeat. *C. elegans* researchers created transgenic models of four possible dipeptides that could potentially lead to toxic cellular consequences. Subsequent characterization revealed that arginine-containing dipeptides exhibited potent motor neuron and muscle cell toxicity (Rudich et al., 2017). A distinct effort involved *C. elegans* overexpressing nine and 29 GGGGCC repeats under the broadly active *hsp-16* promoter. The phenotypes were more severe in animals expressing 29 repeats versus nine repeats and included paralysis followed by lethality (Wang et al., 2018). A forward genetic screen of the 29-repeat transgenic worms identified uncharacterized genes, still under analysis, that reversed this severe phenotype. In addition to overexpressing the hexanucleotide repeat, the *C. elegans* genome has an ortholog of *C9orf72* termed *alfa-1*; a loss-of-function *alfa-1* mutant that exhibits motor neuron degeneration and a motility defect (Therrien et al., 2013). Non-neuronally, these worms have an endocytosis defect that can be partially rescued by introducing wild-type human *C9orf72* (Corrionero and Horvitz, 2018). *C. elegans* *alfa-1* has also been reported to be involved in nutrient sensing and metabolic control (Ji et al., 2020).

Discovery of *SOD1* mutations in 1993 allowed for the initial modeling of ALS (Rosen et al., 1993), even though *SOD1* mutations are now understood to be responsible for only ∼2% of familial ALS (van Damme et al., 2017). As a cytosolic enzyme, SOD1 catalyzes the detoxification of superoxide. There are more than 100 mutant alleles of *SOD1* associated with disease, and most mutations cause a toxic gain of function in motor neurons (Cleveland and Rothstein, 2001). Some of these mutations, such as G85R and G93A, misfold and then eventually aggregate in motor neurons, as well as *in vitro* (Ghosh et al., 2020). Other mutations, such as A4V, cause ER stress, but the underlying mechanism for this stress remains unclear (Perri et al., 2020). Dysfunctional proteostasis correlates with ER stress in a mouse model of G93A SOD1 (Kikuchi et al., 2006); moreover, motor neuron subclasses vulnerable to degeneration also exhibit increased ER stress upon G93A or G85A SOD1 expression (Saxena et al., 2009). Pan-neuronal expression of human G85R SOD1 in *C. elegans* leads to locomotor defects, development of aggregates and axonal abnormalities, such as reduced numbers and diameter of axons and fewer organelles within the remaining axons (Wang et al., 2009). Thompson et al. (2014) demonstrated that co-expression of torsinA, a highly-expressed neuronal chaperone in humans, can attenuate the ER stress caused by human G85R SOD1, as well as restore normal locomotion to transgenic *C. elegans*. Overexpression of a different human SOD1 mutation, G93A, specifically in motor neurons led to age-dependent paralysis as a consequence of axonal guidance defects (Li et al., 2013). Notably, these same two mutations (G85R and G93R) were subsequently modeled as single-copy insertions, along with three other common SOD-1 mutations (A4V, H71Y, L84) in *C. elegans*. The impact on both glutamatergic and cholinergic neuron degeneration was examined for all five of these mutations (Baskoylu et al., 2018). G93R displayed phenotypes consistent with a toxic gain-of-function phenotype in cholinergic neurons, while G85R caused glutamatergic neurodegeneration following exposure to the neurotoxin paraquat (Baskoylu et al., 2018).

Although the associated pathogenic mechanisms have yet to be understood, TAR DNA-binding protein-43 (TDP-43) (Arai et al., 2006) and FUS (Kwiatkowski et al., 2009) have been identified in proteinaceous cytoplasmic inclusions in motor neurons of ALS patients. ALS-causing mutations within the TDP-43-encoding gene, *TARDBP*, are associated with 0.9% of patients, whereas 0.7% of patients harbor mutations in *FUS* (van Damme et al., 2017). TDP-43 and FUS, which are DNA/RNA-binding proteins, regulate transcription, splicing, RNA transport and stress granule formation (Aksoy et al., 2020), and are predominantly nuclear under wild-type conditions. Notably, when mutated, both proteins display cytoplasmic mislocalization, along with protein aggregates, as a hallmark of ALS (Liu et al., 2017).

Multiple research groups have examined the phenotypic consequences of TBP-43 expression within *C. elegans* neurons. For example, pan-neuronal expression of human wild-type TDP-43 under control of the *snb-1* promoter caused slowed and uncoordinated movement, as well as defasciculation of the motor neurons of the transgenic worms (Ash et al., 2010). In a separate effort, using the same pan-neuronal promoter, motility defects, aggregation and neurodegeneration specifically within the GABAergic neurons, resulted from the expression of mutant variants of TDP-43 (Liachko et al., 2013). These TDP-43 models have led to mechanistic insights, including the observation that calcineurin, a phosphatase, removes the pathological C-terminal phosphate from TDP-43 (Liachko et al., 2016). A phenotypic drug screen discovered that α-methyl-α-phenylsuccinimide (MPS) reversed the locomotion defects associated with TDP-43 mutant worms. MPS is the active metabolite of methsuximide, a currently used anti-epileptic drug, suggesting the utility of repurposing this compound in treating TDP-43 proteinopathies (Wong et al., 2018).

The *C. elegans* genome encodes an ortholog of TDP-43, called TDP-1, that is primarily expressed in the nuclei of neurons and body wall muscle cells (Vaccaro et al., 2012a; Zhang et al., 2012). TDP-1 contains most motifs found in human TDP-43, including two RNA-binding motifs, and nuclear import and export signals. However, the glycine-rich domain of human TDP-43 is missing. Nonetheless, there is still functional conservation between these two proteins; specifically, the toxicity phenotypes associated with the *C. elegans tdp-1* mutant can be rescued by expressing wild-type human TDP-43 (Zhang et al., 2012). Deletion of endogenous *tdp-1* could rescue the neurodegeneration associated with overexpression of mutant human TDP-43 in *C. elegans* GABAergic neurons (Vaccaro et al., 2012a).

FUS variants have also been examined in transgenic *C. elegans*. Expressing a FUS variant prone to aggregation in GABAergic neurons via the *unc-47* promoter resulted in neurodegeneration, synaptic dysfunction, paralysis and aggregation, whereas expression of wild-type FUS did not cause these phenotypes (Vaccaro et al., 2012b). In a separate study, when FUS mutants were expressed pan-neuronally under control of the *rgef-1* promoter, multiple mutations associated with aggregation resulted in motor dysfunction, while variants that do not aggregate did not cause observable phenotypes (Murakami et al., 2012). The *C. elegans* genome has an ortholog of *FUS* named *fust-1*. The *fust-1* gene product is well conserved and has been reported to interact with the ortholog of AGO2 in *C. elegans*, ALG-1, a core microRNA-induced silencing complex component. Additionally, FUST-1 is required to achieve maximum microRNA (miRNA)-mediated gene silencing (Zhang et al., 2018).

FTD with parkinsonism linked to chromosome 17 (FTDP-17) is characterized by the accumulation of the microtubule-associated protein tau. Symptoms include neurodegeneration and cognitive decline. However, the underlying mechanisms remain unclear. Researchers have developed transgenic *C. elegans* models expressing either the wild-type human tau or FTDP-17-causing variants (P301L or V337M) under control of a pan-neuronal promoter (Kraemer et al., 2003). Expression of the mutant alleles resulted in cholinergic signaling defects and GABAergic neurodegenerative phenotypes. Additionally, these animals were uncoordinated, displayed egg-laying defects and had a reduced lifespan. Western blotting demonstrated the accumulation of phosphorylated insoluble tau in these animals (Kraemer et al., 2003). Another study showed that expression of either normal human tau or pseudohyperphosphorylated tau within the GABAergic neurons caused uncoordinated movement without neurodegeneration (Brandt et al., 2009). Notably, pseudohyperphosphorylated tau-expressing animals did exhibit a developmental abnormality with visible gaps in the motor neurons.

The tau mutation A152T is a rare risk factor for FTD and AD. Worms with pan-neuronal expression of human tau A152T displayed GABAergic neuron degeneration (Pir et al., 2016). A subsequent study found that pan-neuronal tau A152T expression effectuated the loss of glutamatergic neurons significantly more than GABAergic neurons and attributed this loss to differential vulnerability to excitotoxicity (Choudhary et al., 2018). Results from a genome-wide RNAi screen (Kraemer et al., 2006) and a follow-up study using a *C. elegans* model overexpressing human tau pan-neuronally discovered that the unfolded protein response (UPR) of the ER (UPR^ER^) is a potential modulator of tau proteostasis (Waldherr et al., 2019). Furthermore, the expression of the constitutively active X-box binding protein 1 (XBP-1), the driving transcription factor of the UPR^ER^, improved tau clearance in the cytoplasm and enhanced neuron survival (Waldherr et al., 2019).

### HD

An autosomal-dominant disorder characterized by the progressive and age-associated decline in motor control and cognition, typically beginning in mid-life, HD is a profoundly tragic neurodegenerative disease because of its strong familial association and highly predictive mortality, owing to the absence of any disease-modifying therapy. In humans, HD is characterized by an increase in polyglutamine repeats (polyQs) in the N-terminus of the huntingtin protein (HTT). Repeat length correlates with the age of onset and severity, with 35 repeats being the threshold of disease development (Orr and Zoghbi, 2007). In *C. elegans*, HD has proven archetypical for modeling the protein misfolding pathology and altered proteostasis shared among neurodegenerative diseases.

Despite the absence of a *C. elegans* ortholog of HTT, several groups have generated transgenic animals expressing polyQ tracts and human HTT fragments with varying polyQ-repeat lengths in different neuronal subtypes to study HD pathology (Satyal et al., 2000; Parker et al., 2001; Morley et al., 2002; Nollen et al., 2004). One of these is limited to the ASH sensory neuron, achieved by placing the transgene under the control of the *osm-10* promoter. Degeneration correlates with polyQ length and is readily detected by the failure of this ciliated neuron to uptake a fluorescent dye (Faber et al., 1999). Expression of HTT with normal and expanded polyQ tracts specifically in the touch receptor neurons, under control of the *mec-3* promoter, caused mechanosensory neuron dysfunction and degeneration of axons in a polyQ-length-dependent manner (Parker et al., 2001). These models have been used to investigate the mechanisms underlying HD and the therapeutic potential of various compounds.

Because mouse models of neurodegenerative diseases showed that loss of autophagy enhanced degeneration, researchers tested the effect of neuron-specific autophagy gene loss to evaluate the role of protein turnover in an HD *C. elegans* model. Inactivation of these genes in the presence of polyQ expansions increased the accumulation of aggregates and their toxicity, resulting in augmented neurodegeneration (Jia et al., 2007). Similarly, researchers observed that glucose was neuroprotective in the 128-repeat polyQ HD model. This protection was DAF-16 dependent and glucose operated by reducing misfolded protein levels (Tauffenberger et al., 2012). As a final example, the protective activity of rutin, previously shown to be therapeutic in rat models of HD, was demonstrated through a reduction in polyQ-induced neuron death and elevated activation of DAF-16 (Cordeiro et al., 2020).

### PD

Following AD in prevalence, PD is the second most common neurodegenerative disease, afflicting ∼10 million people worldwide. Unlike HD, PD is primarily idiopathic, with only ∼10% showing familial inheritance (Hernandez et al., 2019). Although early-onset PD accounts for a small amount of cases, the vast majority of individuals display symptomality late in life, typically after age 65, with increased age being the predominantly accepted risk factor. The primary clinical hallmarks of PD include postmortem evidence of Lewy bodies and loss of dopaminergic neurons in the substantia nigra region of the brain. Owing to its central role in PD pathogenesis, α-synuclein (α-syn), which is encoded by the *PARK1/SNCA* locus in humans and is associated with autosomal-dominant PD (Meade et al., 2019), has been modeled extensively in *C. elegans*. α-syn is also a major component of Lewy bodies and multiplication of the *PARK1/SNCA* locus has been linked to enhanced aggregation and dopaminergic neuron death (Mezey et al., 1998; Singleton et al., 2003). *C. elegans* has orthologs of many PARK genes, with the notable exception of *PARK1*/*SNCA*. This conveniently allows *C. elegans* researchers to overexpress α-syn without interference from endogenous α-syn or a dominant-negative effect, as the worm essentially serves as ‘an α-syn null genetic background’. Researchers have developed numerous *C. elegans* models of PD and, in most, α-syn and GFP are selectively expressed from either a dopaminergic or pan-neuronal promoter. The dopamine transporter (*dat-1*) promoter delivers higher expression levels and concomitant dopaminergic neurodegeneration from overexpression of either wild-type or familial mutant forms of α-syn (Lakso et al., 2003; Cao et al., 2005; Karpinar et al., 2009). Worm models of α-syn-induced neurodegeneration have been widely used in combination with genetic and drug screening in a discovery pipeline comprised of more than one model system to successfully demonstrate the evolutionary conservation of function for modifiers and pathways.

Cooper et al. (2006) demonstrated that overexpression of human α-syn in yeast blocks ER-to-Golgi vesicular trafficking; this was the first report of cellular malfunction due to α-syn. Moreover, this study employed multiple model systems to provide additional support with results from *Drosophila*, *C. elegans* and primary rat neuronal cultures confirming that increased expression of a key vesicular trafficking pathway protein, Rab1, could rescue dopaminergic neuron loss induced by α-syn (Cooper et al., 2006). A separate screen of 190,000 compounds for reversal of α-syn-induced yeast toxicity identified N-aryl benzimidazole (NAB). Notably, NAB activity was conserved in neurons, as it protected *C. elegans* dopaminergic neurons and α-syn-expressing rat primary neurons from α-syn-induced neurodegeneration (Tardiff et al., 2013), as it did in dopaminergic neurons differentiated from patient-derived induced pluripotent stem cells (Chung et al., 2013). Another large-scale candidate gene screen conducted in *C. elegans* expressing α-syn::GFP in a *daf-2* receptor-mutant background, a pivotal regulator of aging and lifespan, identified the glycolytic enzyme GPI-1/GPI1 as a neuromodulatory factor. Concurrently, GPI-1/GPI1 was shown to protect against α-syn proteotoxicity in *Drosophila* and mouse primary neuronal cultures (Knight et al., 2014). These collaborative pipeline-type studies emphasize the importance of basic cell biology in neurodegeneration. Furthermore, they exemplify the power of using multiple model systems to identify evolutionarily conserved pathways and new targets as functional effectors of neurodegeneration.

One of the enduring mysteries of PD is the selective vulnerability of dopaminergic neurons to degeneration. It has been hypothesized that dopamine itself may be a key contributor to pathogenesis, as it is more readily oxidized than other neurotransmitters. *In vitro* experiments with purified α-syn polypeptides demonstrated that addition of dopamine enhances and stabilizes the oligomeric state of α-syn, whereas deletion of five amino acid residues (^125^YEMPS^129^) in the C-terminal region of α-syn abolished this effect (Herrera et al., 2008). Using transgenic *C. elegans* expressing the aggregation-prone A53T α-syn variant, researchers investigated the interaction between α-syn and dopamine with and without the dopamine interaction site (Mor et al., 2017). The animals were crossed into an established strain that overproduces dopamine by the overexpression of tyrosine hydroxylase, the product of *cat-2* (Cao et al., 2005), to reveal that excess dopamine exacerbates neurodegeneration. These data corroborated results showing that enhanced dopamine levels in mice expressing human A53T α-syn induced nigrostriatal degeneration (Mor et al., 2017). Importantly, worms expressing A53T α-syn lacking the ^125^YEMPS^129^ sequence exhibited baseline levels of neurodegeneration. This differential effect depended on the presence of *cat-2* overexpression (Mor et al., 2017). The use of *C. elegans* in this study provided mechanistic clarity to help resolve a long-standing question of PD neuropathology with clinical implications.

Mutations in leucine-rich repeat kinase 2 (*LRRK2*) are associated with autosomal dominant PD. Based on biochemical experiments, mutations are linked to modifications in the GTPase and kinase activities of LRRK2 that are likely the basis of the neuronal toxicity in these PD patients. Transgenic expression of wild-type or disease-causing mutant *LRRK2* under pan-neuronal or dopaminergic-specific promoters results in neuronal loss in *C. elegans* (Saha et al., 2009; Liu et al., 2011; Johnson et al., 2015). Using the dopaminergic LRRK2 model, Liu et al. (2011) confirmed the effects of select inhibitors of LRRK2 kinase activity identified from chemical library screening. Additionally, the reduced glutaredoxin (Grx1; also known as GLRX) levels observed in postmortem PD patient midbrain samples was subsequently validated in a *C. elegans* LRRK2 model. Specifically, loss of worm Grx1, combined with LRRK2 overexpression, resulted in significantly greater levels of neurodegeneration (Johnson et al., 2015). While its kinase activity offers an attractive target for drug intervention, LRRK2 is a complex multidomain protein comprised of over 2500 amino acids, and largely remains a functional enigma underlying PD pathology. Interestingly, phosphoproteomic analysis has identified Rab GTPases as biological substrates for LRRK2-dependent phosphorylation (Steger et al., 2016). It is notable that among the Rab proteins modified by LRRK2 (Rab1, Rab3, Rab8, Rab10 and others), several were identified as modulators of α-syn-dependent neurodegeneration using *C. elegans* well over a decade ago (Cooper et al., 2006; Gitler et al., 2008). More recently, a convergence of α-syn and LRRK2 dysfunction around intracellular trafficking has coincided with evidence highlighting the significance of endolysosomal vesicular fusion and lysosome function in PD-associated neurodegeneration (Winckler et al., 2018). In this context, there is increased interest in factors affecting lipid homeostasis as a potential avenue of therapeutic target development (Fanning et al., 2020). *C. elegans* has already been used to demonstrate the neuroprotective effect of stearoyl-CoA desaturase inhibition (Fanning et al., 2019), an effect that translates to human-derived neurons cultured in the presence of neurotoxic α-syn (Vincent et al., 2018). Likewise, phosphatidylethanolamine (PE) deficiency enhances neurodegeneration in transgenic α-syn animals and ethanolamine supplementation reverses this effect (Wang et al., 2014). These data correlate with reports of an age-dependent decline in PE found in PD patients.

The role of the lysosome in misfolded protein degradation (autophagy) and damaged mitochondrial clearance (mitophagy) has emerged as a substantial factor in PD. Again, over a decade ago, *C. elegans* RNAi screening to identify modifiers of α-syn misfolding and neurodegeneration revealed *in vivo* effects of proteins that function in autophagy or mitophagy (ATG7, ULK1, PINK1, PRKN), endolysosomal trafficking (VPS41) and lysosome function (cathepsin D, ATP13A2/PARK9) on dopaminergic neurodegeneration (Hamamichi et al., 2008; Gitler et al., 2009; Qiao et al., 2008). Most significantly, Sidransky et al. (2009) defined an association between PD and mutations in the human glucocerebrosidase gene, *GBA1* (also known as *GBA*), a causal factor in the rare lysosomal storage disorder Gaucher's disease. Whereas homozygous mutations in *GBA1* cause Gaucher's disease, heterozygous carriers are of significantly higher risk of PD. Thus, a deficit in glucocerebrosidase leads to accumulation of glycolipids. Using transgenic *C. elegans* overexpressing human α-syn in the dopaminergic neurons, Mazzulli et al. (2011) reported that neuron-specific RNAi knockdown of *gba-1* enhanced dopaminergic neurodegeneration. This supported an initial hypothesis that α-syn misfolding and GBA-1 loss of function combine in a pathogenic loop of exacerbated neurotoxicity. As of writing, *GBA1* mutations comprise the most common class of genetic cases of PD.

These examples are only a small sampling of the numerous contributions *C. elegans* models have made to neurodegenerative disease research. Through the collective efforts of worm researchers worldwide, functional characterization of previously uncharacterized or poorly understood disease-associated gene products has provided mechanistic insights to illuminate the translational path forward. As discussed below, the toolbox of resources available in the *C. elegans* research community continues to expand, further establishing this model system as a means to enable and accelerate discovery in this area of urgent global need.

## Tools and approaches for the functional characterization of neurodegenerative mechanisms

### Transgenic nematodes

Researchers incorporate exogenous DNA into *C. elegans* for numerous purposes, such as to rescue mutant alleles, analyze gene function, or to examine gene expression (see poster, ‘Transgenic nematode production’). In all cases, the gene of interest is engineered to be controlled by an endogenous *C. elegans* promoter. This promoter permits expression in all or in selected tissues. Additionally, for strain construction, a fluorescent or phenotypic marker (GFP, mCherry, *rol-6*) is also used to distinguish transgenic progeny. For many years, these expression constructs were microinjected, via the germ line, into *C. elegans*, where they become linearized and naturally form concatemers that result in overexpression in the progeny of successfully injected animals (Mello et al., 1991; Berkowitz et al., 2008). A caveat to this method is that it is not possible to control the amount of transgene incorporated into separate transgenic lines. To correct for this, researchers typically analyze multiple lines of transgenic animals and determine a consensus phenotype for transgenic overexpression. More recently, worm researchers have turned to CRISPR/Cas9-guided genome engineering approaches for the generation of transgenic animals. This method can achieve single-copy insertion of transgenes, thus circumventing the issues of overexpression described above. Additionally, if one were to express a human disease mutation in a *C. elegans* strain that was concomitantly null mutant for the same gene, this would result in a ‘humanized’ worm. As an example, Zhu et al. (2020) sought to functionally validate epilepsy-related mutations and generated a strain in which a null mutation was introduced into the *C. elegans* ortholog of *STXBP1*, *unc-18*, via CRISPR/Cas9, which resulted in paralysis. They found that the human ortholog functionally replaced the worm gene and that the epilepsy-associated variants caused seizure-like activity. Although CRISPR/Cas9 and other means by which single-copy insertion or editing can be achieved are often desirable approaches, in the case of neurodegenerative disease modeling, multicopy transgene expression can actually be preferable. For example, dose-dependent neurotoxicity of α-syn and Aβ are established characteristics of human pathology that can be recapitulated in transgenic *C*. *elegans* harboring tandem multicopy arrays. Lower copy number arrays or less robust promoters can result in different levels of neurodegeneration, which is useful for threshold-based genetic screening of suppressors and enhancers, or a complete lack of degenerative phenotype *in vivo* (Harrington et al., 2010; Therrien and Parker, 2014; Wang et al., 2016).

### Forward genetic screening

In the early 1960s, Nobel laureate Sydney Brenner began a nascent interest in developing a model to understand an organism's nervous system in its entirety. He chose *C. elegans* to uncover cellular details of the mechanistic foundation of the nervous system. This nematode was a wise choice because of its ease of genetic manipulation. While males exist among populations as a result of chromosomal non-disjunction events, *C. elegans* is predominantly a hermaphrodite in nature. Thus, for example, if a hermaphrodite has one mutation and is crossed with a male that has a different mutation, the next generation hermaphrodite will inherit both mutations as a heterozygote. Achieving homozygosity is simply a matter of ‘selfing’ this individual hermaphrodite, which will produce up to 300 offspring.

Brenner began understanding the nervous system in *C. elegans* by identifying synaptic transmission genes via a forward genetic screen (see poster, ‘Genetic modifier screens’). He treated worms with ethyl methanesulfonate (EMS) and screened for uncoordinated (or ‘Unc’) worms, identifying 77 Unc genes (Brenner, 1974). Mapping these genes was arduous, and cloning mutations had no guarantee of success. Now, with advances in genome sequencing techniques, there has been a renaissance in the approach. Countless forward genetic screens have been performed, based on individual phenotypes or as modifier screens. As examples, an EMS screen of 80,000 haploid *C. elegans* genomes expressing GFP in the dopaminergic neurons followed by fluorescence-activated sorting of individual worms with defective dopaminergic neuron differentiation, identified 22 alleles organized into six complementation groups. It was estimated that the screen reached ∼78% saturation based on the allele recovery rate (Doitsidou et al., 2008). In a separate effort, following the development of *C. elegans* models of tauopathy by pan-neuronally expressing wild-type or FTD-associated mutant tau (tau-FTDP-17) (Kraemer et al., 2003), forward genetic screens identified two new molecular factors, SUT-1 and SUT-2, that participate in the activation of tau (Kraemer and Schellenberg, 2007; Guthrie et al., 2009). SUT2 is now an established susceptibility factor for tau pathology in the mammalian brain (Wheeler et al., 2019). Worms harboring deletions and point mutations that have been isolated from genetic screens and carefully annotated are deposited in the *Caenorhabditis* Genetics Center, with over 20,000 strains available to the research community. These include mutations in many genes associated with neurodegenerative diseases.

### Reverse genetic screening

Neurodegeneration-associated phenotypes have also served as the basis of reverse genetic screens (see poster, ‘Genetic modifier screens’). The discovery and application of RNAi has revolutionized phenotypic analysis in *C. elegans*. Unlike in most animals, RNAi is typically performed by feeding parental *C. elegans* with bacteria that produce double-stranded RNA (dsRNA) against the target of interest (Timmons et al., 2001). The progeny exhibit altered phenotypes that are scored. RNAi feeding of individually cloned dsRNA of nearly every predicted open-reading frame has been used in high-throughput screening of embryonic and post-embryonic phenotypes in *C. elegans* (Kamath et al., 2001, 2003). As an example, an RNAi screen covering 85% of the genome was performed in transgenic worms that express normal and FTDP-17 mutant human tau display uncoordinated phenotypes (Unc). Notably, 75 genes enhanced the Unc phenotype. Among these, the 46 candidates with human homologs were preferentially examined further and could be broadly classified as genes involved in dysregulated cell signaling (Kraemer et al., 2006).

### RNAi in neurons

Historically, experimental and anecdotal reports suggested that RNAi was less effective in neurons (Asikainen et al., 2005), although mutant worm strains with enhanced sensitivity to RNAi, such as *rrf-3*, *lin-15b* or *eri-1*, bypass this limitation (Simmer et al., 2002; Kennedy et al., 2004). However, when performing RNAi in these strains, the silencing is systemic and occurs in all tissues of the animal. An alternative method for neuronal RNAi involves selective expression of *sid-1*, which encodes the ATP-independent dsRNA transporter (see poster, ‘Genetic modifier screens’). This is accomplished by generating *sid* mutant animals expressing *sid-1* under the control of a neuron-specific promoter that restricts RNAi to neurons. Calixto et al. (2010) first used this approach to demonstrate successful selective pan-neuronal knockdown of targets. Similar strains have been developed for dopaminergic or glutamatergic neuron-specific RNAi (Harrington et al., 2012; Griffin et al., 2019). Using a neuronal-specific RNAi strain (versus *eri-1* or *rff-3*) is an important strategy for studying the effects of depleting essential gene targets that would be lethal for *C. elegans* if knocked down systemically.

Comprehensive libraries of individual RNAi ‘feeding vectors’ are available from multiple sources, enabling large-scale screening efforts in neuronal contexts (Kamath et al., 2003; Rual et al., 2004). For example, a genome-wide RNAi screen for neurodegenerative phenotypes utilized a transgenic *C. elegans* strain in which human mutant TDP-43 was expressed pan-neuronally and caused motor neuron dysfunction. The authors identified 46 genes (out of 16,767 screened) that, when knocked down, partially suppressed the TDP-43-induced motor phenotype (Liachko et al., 2019). They then focused their efforts on the 24 candidates with human homologs. Knocking down one of these candidates, glucuronic acid epimerase (GLCE), in human cultures protected against the phosphorylated TDP-43 accumulation caused by oxidative stress. Additionally, GLCE expression was significantly decreased in the brains of patients with frontotemporal lobar degeneration with TDP-43 inclusions (FTD-TDP) relative to normal controls (Liachko et al., 2019). This exemplifies the value of *C. elegans* as a means to accelerate the translational path through defining previously undiscovered genetic modifiers, susceptibility factors, or potential therapeutic targets with direct disease relevance.

## Strategies to examine neurodegeneration

The pathological loss of neurons in neurodegenerative diseases is recapitulated in *C. elegans* models, where conditions that promote either neuron survival or loss can be assessed in live animals that express fluorescent proteins in the neuronal class of interest (see poster, ‘Strategies to examine neurodegeneration’). Neurodegeneration is induced by expressing pathogenic proteins within specific the neuronal class(es), followed by scoring subtle attributes of neuronal health, including cell body rounding (a sign of apoptosis or necrosis), the disappearance of axons and/or axonal blebbing, broken neurites and/or retreat of dendritic terminals. Given the limited numbers of neurons comprising specific classes in *C. elegans*, researchers can score neurodegeneration with unparalleled accuracy in hundreds of animals per condition to provide robust and rigorous analyses.

### Genetic crosses to examine neuronal phenotypes

To tackle familial forms of neurodegenerative disease, *C. elegans* investigators often take a genetic approach by examining mutant variants of endogenous proteins. As previously discussed, a form of familial AD results from mutations in *PSEN1* or *PSEN2*. When mutated, the *C. elegans* presenilin ortholog, *sel-12*, causes ER Ca^2+^ dysregulation similar to that found in mammals (Sarasija and Norman, 2015). Notably, further research with the *sel-12* mutant revealed mitochondrial morphological and metabolic phenotypes that fostered neurodegeneration (Sarasija et al., 2018). Another *sel-12* mutant phenotype, an egg-laying defect, can be rescued by mutating the worm *REST* ortholog, *spr-4* (Lakowski et al., 2003). These *spr-4* mutations, originally isolated from a forward genetic screen for egg-laying defects, have since revealed insights into pathways regulating neurodegeneration. For example, mutations in SPR-4/REST enhance Aβ-induced glutamatergic neurodegeneration in transgenic *C. elegans*; this result correlated with those from companion studies in mammals (Lu et al., 2014).

Notably, mutations in *C. elegans pink-1* and *pdr-1*, which are homologous to the recessive familial PD proteins PINK1 and PRKN, do not display neurodegenerative phenotypes even though they are null and strong loss-of-function mutations, respectively. However, these genetic lesions do cause dopamine-dependent behavioral deficiencies (Cooper et al., 2017) that could potentially be used in screens for gene-by-gene or gene-by-environment interactions. As examples, exposing *pdr-1* mutant worms to either a secondary stressor such as α-syn, a proteasome inhibitor (i.e. MG-132) or the neurotoxic dopamine analog 6-hydroxydopamine (6-OHDA) enhances neurodegeneration (Martinez et al., 2015; Hartman et al., 2019). Conversely, *pink-1* mutant worms in which α-syn is expressed in the dopaminergic neurons have significantly enhanced neurodegeneration, but no sensitivity to the proteasome inhibitor MG-132, and exhibit reduced neurodegeneration upon 6-OHDA exposure (Martinez et al., 2015; Hartman et al., 2019). These studies highlight the ability to dissect mechanisms in a genetically tractable organism as an informative prelude to more expensive and time-consuming validation studies in mammalian models.

### Modeling protein misfolding

Many researchers also want to establish whether neurodegeneration is associated with alterations in protein handling. However, the small size of nematode neurons render visual inspection of protein misfolding a challenge and impractical for screening or serial analyses. To circumvent this limitation, models of pathogenic proteins conjugated to fluorescent molecules have been expressed in the large body wall muscle cells in *C. elegans*. This enables researchers to assess changes due to alterations in protein misfolding, apparent aggregate density or count following RNAi or small-molecule exposures, in distinct genetic backgrounds and over the course of aging. Although these are not neurodegenerative readouts, the ease with which rapid visual screening can be conducted in muscles has afforded researchers a means toward preliminary identification of putative functional modifiers of pathogenic proteins that can be subsequently investigated in neurons and/or mammalian cells. As outlined in the following sections, this strategy has identified multiple gene and drug modifiers of neurodegeneration that have successfully predicted outcomes in mammalian models.

As many neurodegenerative diseases have concomitant defects in protein handling, *C. elegans* researchers have developed models of pathogenic protein misfolding (i.e. Aβ_1-42_, polyQ_n_, tau and α-syn) in the nematode body wall muscle cells (see poster, ‘Strategies to examine neurodegeneration’). Misfolded protein density and/or count of aggregated pathogenic proteins can be assayed in the α-syn models (Hamamichi et al., 2008; van Ham et al., 2008). Following hypothesis-based bioinformatics approaches to systematically define candidate targets potentially associated with PD, RNAi was used to identify genetic modifiers of α-syn::GFP misfolding and accumulation in screens of hundreds of candidate targets, initially in body wall muscle cells (Hamamichi et al., 2008; Knight et al., 2014). Upon a winnowing of candidates, the significantly shorter lists of hits were then examined for knockdown or overexpression in dopaminergic neurons (Hamamichi et al., 2008; Knight et al., 2014). Using this tiered strategy in screens for α-syn-related phenotypes, neuroprotective candidates identified in *C. elegans* have been repeatedly validated in mammals and/or in human genetic studies. For example, overexpression of the lysosomal trafficking protein VPS41 (VPS-41 in *C. elegans*) protects cells against several PD-related neurotoxins, including 6-OHDA and rotenone (Ruan et al., 2010). Moreover, overexpression of VPS41 decreases the levels of α-syn protein levels in human neuroglioma cells (Harrington et al., 2012). Considering the pathological overlap between PD and AD, VPS-41 was examined for therapeutic potential using *C. elegans* Aβ paralysis and neurodegeneration models. Notably, VPS-41 protected in both diseases, but with notable functional distinctions between the modes of neuroprotection between PD and AD. Specifically, in the α-syn model, neuroprotection is mediated via RAB-7 and AP-3, while in the Aβ model, it occurred through an ARF-like GTPase gene product, ARL-8 (Griffin et al., 2018). Given the high conservation of endocytic components among cells and species, the ability to dissect such mechanistic distinctions in neuroprotective pathways using *C. elegans* warrants further consideration when prioritizing targets for *in vivo* modeling.

In the Aβ models of AD, Aβ peptide accumulation in muscle cells induces paralysis, which can be readily quantified. Two different muscle expression models are widely used in the field; one constitutively expresses Aβ_3-42_ and is utilized for studies of toxicity and metabolism (Link, 1995), while the other provides inducible expression of Aβ_1-42_ (Link et al., 2003). RNAi screens can identify the targets of drugs previously shown to inhibit Aβ aggregation. Studies of the antioxidant resveratrol in *C. elegans* have demonstrated that it is neuroprotective and prevents the formation of Aβ aggregates (Regitz et al., 2016). Following verification of this protection in *C. elegans* constitutively expressing Aβ_3-42_ in the body wall muscles, RNAi was performed on proteostasis-related targets. Notably, UBL-5, part of the mitochondrial UPR (UPR^mt^), was identified as a critical component of resveratrol-mediated aggregate prevention and XBP-1, a key regulator in the UPR^ER^, was also necessary (Regitz et al., 2016).

The polyQ_n_ HD body wall muscle expression models can provide both quantitative and behavioral information. Aggregate accumulation positively correlated with adverse effects on motility, which worsened with increasing polyQ lengths (Q29 versus Q35 versus Q82) (Satyal et al., 2000). These models have been used in concert with either α-syn or Aβ models in comparative RNAi screens to parse gene candidates into subcategories for those that have a common basis in proteostasis malfunction versus those that drive a more pathogenic-protein specific misfolding event (Nollen et al., 2004; van Ham et al., 2008; Knight et al., 2014). For example, a genome-wide RNAi screen performed on animals expressing polyQ expansions identified genes involved in RNA processing and in the synthesis, folding, trafficking and degradation of proteins as important in polyQ aggregate formation (Nollen et al., 2004). A subsequent screen using animals expressing misfolded α-syn identified ER/Golgi vesicle-trafficking and quality control genes (van Ham et al., 2008).

### Chemical modulation of neurodegeneration

A variety of chemical compounds can alter the extent of neurodegeneration in *C. elegans* (see poster, ‘Strategies to examine neurodegeneration’). Neurodegeneration enhancers such as 1-methyl-4-phenyl-1,2,3,6-tetrahydropyridine (MPTP) and 6-OHDA induce PD-like phenotypes in *C. elegans*, rodents and other model organisms (Betarbet et al., 2002; Braungart et al., 2004; Cao et al., 2005). Conversely, chemical modulators can also attenuate neurodegeneration through distinct mechanisms. Bafilomycin, for example, reduces neurodegeneration by inhibiting autophagic vesicle maturation (Pivtoraiko et al., 2010), dantrolene reduces neurodegeneration through inhibition of intracellular calcium release in the ER (Xu et al., 2001), and probucol attenuates neurodegeneration via its antioxidant properties (Ray et al., 2014). Diverse modulators, applied alone or in tandem with transgenes, coupled with medium- or high-throughput screening, facilitate the discovery of drugs to combat neurodegenerative diseases.

In addition to the aforementioned examples, a transgenic *C. elegans* ALS model expressing mutant *TARDBP* was an initial platform for screening chemical compounds; the hits, neuroleptics, were then validated in a zebrafish model, and the most potent molecules were subsequently examined in mice and in a small clinical trial (Patten et al., 2017). This screen is among the first in the neurodegenerative disease class to realize the true translational potential for target engagement in humans. In another example, after screening >14,000 chemical candidates that reduce aggregation and fibril formation in a human neuroblastoma cell line (SH-SY5Y), a hit compound also reduced α-syn-induced neurodegeneration in *C. elegans* (Pujols et al., 2018). The predictive nature of these screening results, based on genomic and cellular homology across species, demonstrates the utility of this nematode as part of a comprehensive discovery scheme.

### Monitoring neuronal cell health

The transparent nature of *C. elegans* is useful for gauging cellular health via fluorescent reporters (see poster, ‘Strategies to examine neurodegeneration’). As both autophagy and mitochondrial maintenance or impairment have emerged as pivotal to disease and aging (Chan, 2006; Malpass, 2013), several such reporters inform cellular health within the context of neurodegenerative diseases.

The autophagy marker LGG-1 [the worm homolog of LC3 (also known as MAP1LC3A)] is expressed in the muscles, intestine, pharynx, vulva, hypodermis, somatic gonad and nervous system of *C. elegans*, and allows detection of autophagosomes using the fluorescent reporter GFP::LGG-1 (Palmisano and Melendez, 2016). This marker shows diffuse intracellular expression under baseline conditions, with increased expression becoming evident in conditions that induce autophagy, such as starvation. Monitoring the localization and/or intensity of GFP::LGG-1 can query gene targets that functionally affect autophagy. Another transgenic line to evaluate mitophagy consists of the worm ortholog of human mitophagic regulator BNIP3, DCT-1, tagged with GFP along with dsRed::LGG-1 (Palikaras et al., 2015). DCT-1::GFP localizes to the outer mitochondrial membrane and dsRed::LGG-1 labels autophagosomes. Mitophagy-inducing stimuli can be visualized by the colocalization of these markers. Likewise, mitochondrial fragmentation can be visualized by expressing TOMM-20::mCherry in *C. elegans* (Jiang et al., 2015). The localization of the mitochondrial import receptor TOMM-20 to the outer mitochondrial membrane allows researchers to study conditions that interrupt the fusion/fission balance.

Assays can quantify mitochondrial stress response through the activation of the UPR^mt^. This mitochondrial quality control program is activated during periods of stress to promote survival and recovery of mitochondrial function. One response of this pathway is the induction of the chaperone HSP-6. By tracking the fluorescence intensity of an *hsp-6*::GFP transcriptional reporter, researchers can measure the activation of UPR^mt^ (Haynes et al., 2007). This readout has proven key to deciphering the impact of the UPR^mt^ in neurodegenerative disease. Notably, in transgenic *C. elegans* PD models, α-syn overexpression appears to co-opt this pathway from a normally protective response to transient stressors into a potential contributor to proteostatic collapse and neurodegeneration when chronically activated (Martinez et al., 2017).

*C. elegans* research ushered in the revolution in live imaging with the Nobel prize-winning report of GFP by Marty Chalfie and colleagues (Chalfie et al., 1994) and the toolbox of *in vivo* reporters of bioactivity for worm research has always been substantial, as the examples discussed here highlight. With the advent of CRISPR/Cas9 editing, novel optogenetic reporters, increasingly diverse indicators and high-resolution microscopy methods, *C. elegans* is poised to remain at the forefront of technologies that can evaluate neuronal function and health in disease modeling.

## Evaluation of neurobehavior

Researchers have developed several common assays with relevance to neurodegenerative disorders that we describe here (see poster, ‘Representative neurobehavioral assays’). For many, automated image software programs assist with accuracy. It should be noted that this section is not a comprehensive survey, as we are unable to describe all the variations and related assays in this space. For a complete review and applications of worm behavioral assays and detailed protocols go to www.wormbook.org.

### General neuronal dysfunction assay

The thrashing assay is a basic assay for generalized neuronal dysfunction. Worms placed in liquid rapidly initiate a response termed ‘thrashing’; a thrash is defined as a directional change in mid-body bending. During the thrashing assay, the frequency of lateral swimming is measured over a short time (30-60 s) for each animal. This assay can be used as a functional readout of neuronal health of transgenic and drug-treated worms. Kraemer et al. (2003) used thrashing assays to discern behavioral distinctions between normal and mutant forms of human tau linked to FTDP-17 expressed pan-neuronally in *C. elegans.* The FTDP-17 variants exhibited decreased thrashing independent of tau expression levels. Similarly, another *C. elegans* model in which neurodegeneration was induced via GABAergic neuron expression of arginine-rich dipeptide repeat proteins designed to functionally mimic the effects induced by such repeats in the human C9orf72 protein, a prevalent cause of ALS and FTD. The altered motility in these animals was associated with dipeptide-repeat expression and correlated with morphological changes in neuron structure indicative of neurodegeneration (Rudich et al., 2017).

### Motor neuron dysfunction

One convenient tool for the identification of genes that code for synaptic transmission regulators is the quantification of sensitivity to aldicarb, an acetylcholinesterase inhibitor. Beginning with the seminal research of Rand and Russell (1984), aldicarb has been used to define many conserved components of cholinergic neurotransmission. Aldicarb causes paralysis in wild-type *C. elegans*, while animals with mutations in acetylcholine neuromuscular signaling at the synaptic cleft are resistant. In contrast, some animals might be more sensitive to aldicarb, indicating increased acetylcholine release. Worms are exposed to aldicarb on a Petri dish, and populations are assessed for paralysis every 10-30 min for a total of 150 min. A study of the neurotoxic selectivity of perfluorooctane sulfonate, a chemical widely used in industry, showed that exposure causes dopaminergic neuron deficits and, through the use of aldicarb, found that this was independent of acetylcholine function (Sammi et al., 2019). In another study, an aldicarb-based assay showed that DNJ-14, a worm homolog of the human cysteine-string protein (DNAJC5), a co-chaperone necessary for synaptic maintenance, was not required for neuromuscular transmission in aging (Mulcahy et al., 2019). Conversely, in an ALS/FTD model, Vaccaro et al. (2012a,b) showed that transgenic worms expressing either mutant or wild-type human TDP-43 and FUS differed in their response to aldicarb, with the mutants exhibiting hypersensitivity to paralysis. In this example and others, aldicarb assays provide a means to correlate behavioral deficits with neurodegeneration.

### Dopaminergic neuron dysfunction

Wild-type *C. elegans* display a dopamine-mediated slowing of locomotion upon entry into a bacterial lawn, owing to a mechanosensory process that detects textural change (Sawin et al., 2000). This dopamine-regulated behavior is termed the basal slowing response (BSR) and is measured by counting the number of body bends in 20-s intervals. Rates are compared between worms placed on plates with food (*Escherichia* *coli*) versus rates of the same worms on plates without food. This method is a phenotypic readout to assess functional changes in dopaminergic neurons. Experiments typically compare the change in body bends over time to positive control worms that do not slow in the presence of food, such as *cat-2* mutants, which lack tyrosine hydroxylase, the rate-limiting enzyme for dopamine synthesis. The BSR assay can elucidate pathways leading to the dysfunction and death of dopaminergic neurons when exposed to toxic substances. As examples, lead (Pb^2+^) exposure increases dopaminergic neurotoxicity in wild-type *C. elegans*, but protein kinase (*pkc*) mutant animals are resistant, an effect that can be quantified via the BSR assay (Akinyemi et al., 2019). Similarly, BSR assays have revealed that Akt signaling provides resistance to manganese-induced dopaminergic neurotoxicity (Peres et al., 2019). This assay is also informative when analyzing the contributions of candidate genes to dopaminergic function. For example, the BSR assay was used to reveal that depletion of the *C. elegans* RAC1 GTPase CED-10, in the presence of α-syn overexpression in dopaminergic neurons, contributes to dopaminergic dysfunction (Kim et al., 2018b). Importantly, BSR defects precede neurodegeneration in *C. elegans* PD models (Martinez et al., 2017); the influence of temporal factors mediating the aging process can thus be evaluated for their progressive impact on dopaminergic neurons over time (Gaeta et al., 2019).

### Glutamatergic neuron dysfunction

A wild-type hermaphrodite *C. elegans* has six sensory neurons spanning its length that detect light touch as administered by stroking the animal with an eyebrow hair glued to a toothpick. This is a mechanosensory response where, normally, animals will exhibit either forward or backward reversal in locomotion when stroked toward the posterior or anterior, respectively (Goodman and Sengupta, 2019). Touch neuron degeneration results in a mutant mechanosensory (Mec) locomotive phenotype with defective forward or backward, or both, movement indicative of glutamatergic dysfunction. As previously described, this assay was used as a readout to examine phenotypic rescue of Aβ-induced degeneration of glutamatergic neurons in the presence of different isoforms of human APOE (Griffin et al., 2019). As another example, animals treated with quinolinic acid exhibited neurodegeneration due to glutamatergic neurotransmission defects (da Silveira et al., 2018). Similarly, in single-copy knock-in SOD1 models of ALS, loss of *sod-1* function produced defects in light touch response indicative of a disruption in glutamate signaling (Baskoylu et al., 2018).

## Correlation between neurodegeneration and aging

Assays that assess whether a treatment or condition affects lifespan and/or healthspan are well established in the field (see poster, ‘Neurodegeneration and aging’). Lifespan consists of healthspan and gerospan. Long-lived animals experience a frailty period termed gerospan, whereas healthspan is defined as the period when an animal has greater than 50% maximal functional capacity (Bansal et al., 2015). Considering that aging is the main risk factor for AD, PD and other neurodegenerative diseases, lifespan and healthspan benchmarks of animal health are appropriate companion assays. Aging-associated molecular signatures, such as epigenetic changes, telomere shortening, proteostasis inhibition, mitochondrial dysfunction, altered lipid metabolism and nutrient sensing dysregulation, among others, can be assessed (Lopez-Otin et al., 2013). Notably, neurodegeneration is associated with many of these cellular processes, and the intersection of aging and neurodegeneration can often be efficiently examined using *C. elegans* models (Knight et al., 2014).

In mammals, the lengthy time requirements to evaluate age-associated changes can be considered a hindrance to higher-throughput analyses. In contrast, the average ∼20-day lifespan of the *C. elegans* wild-type strain Bristol N2 accelerates the discovery of fundamental modulators of neurodegenerative processes within the context of lifespan. A common misconception is that the short survival of *C. elegans* precludes its utility in translating lifespan modifiers in this animal to mammals. A variety of mutants and chemical modifiers of aging have been identified that directly affect highly conserved metabolic pathways and mechanisms shared among metazoans. The most notable example is the *daf-2*/insulin-like signaling pathway (Martins et al., 2016), whereby *C. elegans* research has led to the discovery of critical modulators of cellular and organismal health (i.e. mTOR, Nrf2, FOXO/DAF-16). Among the biological modulators that can be readily examined in *C. elegans* are genetic and epigenetic modifiers, chemical modifiers, microbiome exposures and putative environmental toxicants, in addition to exercise and diet.

Lifespan is assessed by the cohort survival assay (Lionaki and Tavernarakis, 2013), in which at least 120 synchronized L4-stage worms are grown at 20°C and scored for survival/death every 24 h by gently tapping with a platinum wire at both head and tail. A log-rank (Mantel–Cox) test is used to compare survival between strains. Investigation of the role of nicotinamide-N-methyltransferase (NNMT) found that expression of the *C. elegans* homolog, ANMT-1, in dopaminergic neurons increased neuron survival and overall lifespan through the regulation of autophagy (Schmeisser and Parker, 2018). A second study showed that the transcription factor SPR-4/REST regulates aging by reducing neural activity and maintaining neural homeostasis (Zullo et al., 2019). Reproducibility of lifespan data is contingent on rigorous experimental design and repetition. To evaluate variability in quantifying lifespan, Lucanic et al. (2017) reported results from a coalition of multiple laboratories termed the *Caenorhabditis* Intervention Testing Program that assessed longevity for 22 strains, inclusive of three *Caenorhabditis* species. Multiple replicates were collected from three independent laboratories to reveal that diversity in replicate data within a single laboratory for a given strain was more common than variation in lifespan determined across different laboratories. This study highlights the importance of tightly controlled experiments and internal consistency in the replication of lifespan data.

### Healthspan

Like humans, *C. elegans* exhibits a decline in physical ability with age and loss of ability to recover from stress, which manifest as reduced body movement and increased sensitivity to heat and oxidative stress, respectively (Bansal et al. 2015). When searching for genetic modifiers, increased healthspan should be prioritized because long-lived mutants also have increased gerospan (Bansal et al., 2015), which is counterintuitive to enhancing the quality of life. Multiple assays can be performed to assess healthspan. For a comprehensive review, see Bansal et al. (2015). For example, an automated digital video microscopy system examines the average distance *C. elegans* travel on solid agar (Hahm et al., 2015). In a second assay, healthspan can be examined in liquid media. Using this method, researchers learned that *C. elegans* models of neurodegenerative disease had better outcomes following swim training exercise, which improved their neuronal healthspan (Laranjeiro et al., 2019). A third healthspan assay consists of oxidative stress analysis. Worms are transferred to plates containing paraquat, and stress-resistant survival is recorded every 5 days until death (Bansal et al., 2015). Using this assay, it was discovered that a gene product associated with cholesterol metabolism that was neuroprotective in a *C. elegans* PD model maintained healthspan but did not extend lifespan (Zhang et al., 2017).

Addressing lifespan and healthspan, along with other cellular signatures such as mitochondrial and/or ER function and stress responses, as well as alterations in proteostasis and transcriptional or macromolecule profiling (RNA/DNA/miRNA/lipids), provides the *C. elegans* researcher with an arsenal of complementary approaches with which to investigate neurodegenerative diseases. As noted below, the ‘big data’ increasingly emerging from human genomic analyses provides a greater impetus for the application of a more rapid and cost-effective system whereby the significance of genetic variation can be discerned for its impact not only on disease, but also on quality of life.

## Concluding remarks

Among the largely untapped potential avenues of *C. elegans* models of neurodegeneration is the functional annotation of genomic variation among humans to discern factors that confer either susceptibility or resilience. The ever-expanding databases of human sequence information have resulted in an informational overload of variants of uncertain significance (Cooper, 2015). Characterizing these and other changes via functional analyses with the bioassays outlined in this article could greatly advance our understanding of therapeutic options. While it may superficially appear illogical to use an invertebrate such as *C. elegans* for human personalized medicine, there are successful examples of highly specialized drug discovery tailored to the genomic composition of cancer patients (Bangi et al., 2019). *C. elegans* is also being used in the drug discovery efforts for rare glycosylation disorders, N-glycanase 1 (NGLY1) deficiency and phosphomannomutase 2 (PMM2) deficiency (Iyer et al., 2019a,b).

In closing, this article prompted us to reflect on a Primer that we had the privilege of co-authoring for the inaugural issue of Disease Models & Mechanisms. At the inception of this journal as a platform to communicate the increasingly important role of model systems in advancing disease research, we titled our article ‘Traversing a wormhole to combat Parkinson's disease’ (Caldwell and Caldwell, 2008). The choice of this celestial analogy now appears prescient in capturing the exceptional contributions of *C. elegans* models in rapidly advancing discoveries from the experimental space through to the other side of clinical investigation. We contend that the expedience, accuracy, molecular tools and mechanistic capacity of *C. elegans* present an attractive, bioethical alternative to more costly, time-consuming and sometimes redundant *in vitro* or mammalian models. With an increasingly strong track record of translational outcomes that continue to emerge from *C. elegans* research, this microscopic nematode is positioned to help fill the remaining black holes in our understanding of neurodegeneration. Indeed, the gravity of the societal burden from these diseases is worldwide, and weighs on tens of millions of individuals each day. A sense of urgency and innovative strategies that coalesce into model systems research are essential to accelerate discovery and progress. In the words of an admired and fearless explorer known to go where no one (or no worm) has gone before, “Warp speed ahead. Engage!” (Jean-Luc Picard).

## At a glance

A high-resolution version of this poster is available for downloading in the online version of the article at http://dmm.biologists.org/content/13/10/dmm046110/F1.poster.jpg.
